# Modified underwater endoscopic mucosal resection for intermediate-sized sessile colorectal polyps

**DOI:** 10.3389/fmed.2023.1200145

**Published:** 2023-06-20

**Authors:** Dong Hyun Kim, Seon-Young Park, Hye-Su You, Yong-Wook Jung, Young-Eun Joo, Dae-Seong Myung, Hyun-Soo Kim, Nah Ihm Kim, Seong-Jung Kim, Jae Kyun Ju

**Affiliations:** ^1^Division of Gastroenterology and Hepatology, Department of Internal Medicine, Chonnam National University Hospital and Medical School, Gwangju, Republic of Korea; ^2^Department of Pathology, Chonnam National University Hospital and Medical School, Gwangju, Republic of Korea; ^3^Division of Gastroenterology and Hepatology, Department of Internal Medicine, Chosun University Hospital, Gwangju, Republic of Korea; ^4^Division of Colorectal Surgery, Department of Surgery, Chonnam National University Hospital and Medical School, Gwangju, Republic of Korea

**Keywords:** colonic polyps, endoscopic mucosal resection, endoscopy, water, neoplasm (MeSH term)

## Abstract

**Introduction:**

Underwater endoscopic mucosal resection (UEMR) is effective for treating intermediate-sized colorectal polyps. However, it is sometimes difficult to obtain visibility in underwater conditions.

**Methods:**

This prospective, observational, single-center study included consecutive patients with intermediate-sized (10–20 mm) sessile colorectal polyps. Modified UEMR method was used to initially snare the lesion without injection or water infusion. Thereafter, water was infused until the lesion was submerged, then it was resected using electrocautery. We also evaluated the rates of complete resection and procedure-related complications.

**Results:**

Forty-two patients with 47 polyps were enrolled in the study. The median procedure time and fluid infusion were 71 s (42–607) and 50 mL (30–130), respectively. The rates of R0 resection and *en bloc* resection were 80.9 and 97.9%, respectively, with 100% technical success. R0 resection was observed in 42.9% of polyps sized ≥15 mm and 87.5% sized <15 mm (*p* < 0.01). Muscle entrapment was found in 71.4% of patients with polyps sized ≥15 mm and 10% <15 mm (*p* < 0.01). Immediate bleeding occurred in 12.8% of cases and was controlled using a snare tip or hemostatic forceps. Snare-tip ablation and hemostatic forceps ablation were performed in 27.7 and 6.4% of patients, respectively. No delayed bleeding, perforation, or any other complications were reported.

**Conclusion:**

Modified UEMR can be used in cases in which securing visibility or performing the existing UEMR is challenging. Careful treatment is required when removing polyps >15 mm in size.

## 1. Introduction

The current guidelines of the European Society of Gastrointestinal Endoscopy (ESGE) recommend cold snare polypectomy (CSP) for sessile polyps sized 6–9 mm, while hot snare polypectomy (HSP) (with or without submucosal injection) is suggested for the removal of sessile polyps sized 10–19 mm (1). Recently, various methods of polyp resection, such as conventional endoscopic mucosal resection (CEMR), cold snare polypectomy (CSP), and underwater endoscopic mucosal resection (UEMR), have been explored as methods for removing an intermediate-sized polyp (2, 3).

The use of CSP as a removal method for lesions larger than 10 mm is increasing (3–5). CSP can be performed easily; however, there is a high recurrence rate when the histological resection margin is not adequately ablated, and immediate bleeding is relatively common.

The ESGE does not recommend the use of hot biopsy forceps because of high rates of incomplete resection and high risks of adverse events in deep thermal injury and delayed bleeding (1). However, the ESGE suggests HSP (with or without submucosal injection) for the removal of sessile polyps 10–19 mm in size. In most cases, deep thermal injury is a potential risk; thus, submucosal injection prior to HSP should be considered. HSP after injection is described as conventional endoscopic mucosal resection.

Endoscopic submucosal dissection (ESD) shows higher R0 resection and *en bloc* resection compared to EMR; nonetheless, it is more technically difficult and time-consuming (6, 7). Therefore, in the ESGE guideline, ESD is suggested as a standard treatment only for the treatment of polyps larger than 20 mm. On the other hand, the Korean guidelines do not recommend the endoscopic resection method based on the size of the polyp. However, in the case of early cancer, performing ESD is recommended as it can increase the rate of R0 resection and *en bloc* resection (8, 9).

According to a recent study, the rate of complete resection was higher with UEMR than with CEMR for polyps sized 10–20 mm (2). The procedure time was not significantly different from that of CEMR, and the complication rate was not higher than that of CEMR.

In general, UEMR is performed after infusing approximately 200–1,000 mL of water (2, 10, 11). There has been a reported case of hyponatremia when UEMR was conducted for a duodenal lesion (12). Moreover, observing polyps underwater can sometimes be challenging owing to the presence of a mixture of injected water and remnant feces in the large intestine, which can cause blurred vision (13). In some cases, the large intestine contracts, interfering with securing vision. Further, identifying lesions on the folds may require air insufflation (11).

Therefore, we developed a new endoscopic resection method that can compensate for the shortcomings of the existing endoscopic resection method. This hybrid technique was devised for intermediate sessile polyps and involves initially snaring the lesion in air, followed by filling the lumen with water and removing the lesion using electrocautery. We evaluated the effectiveness and safety of this method.

## 2. Patients and methods

### 2.1. Study population

We conducted a prospective, observational pilot study that included consecutive patients undergoing endoscopic resection of sessile colorectal mucosal lesions [adenoma, intramucosal adenocarcinoma, or sessile serrated adenoma/polyp (SSAP)], 10–20 mm in diameter. Endoscopic diagnosis of mucosal lesions was based on their macroscopic appearance. The exclusion criteria were as follows: (1) age < 20 or > 75 years, (2) poor general condition (American Society of Anesthesiologists Physical Status Classification IV or higher), (3) uncontrolled hemorrhagic predisposition, (4) cirrhosis, (5) undergoing dialysis, and (6) pregnant women. All patients were inquired about their current intake of anticoagulant medication. Anticoagulant and antiplatelet agent therapy was discontinued for endoscopic resection according to the recommended cessation period (14). The subjects also underwent various laboratory tests, including complete blood count, blood chemistry tests, prothrombin time, and international normalized ratio.

Morphology was investigated based on the Paris classification (15). Polyp size was initially evaluated by visual estimation and confirmed by comparison with an opened snare (Lariat^®^ 00711119, STERIS^tm^, OH, United States, diameter 10 × 28 mm). In cases where sessile polyps sized <10 mm were observed, polyps sized 4–9 mm were removed by CSP, and those sized 1–3 mm were removed using cold biopsy forceps. If a pedunculated polyp <20 mm in size was found, it was removed with CEMR. However, for polyps sized >20 mm, the procedure was performed by a reservation at a later date.

The primary endpoints were *en bloc* resection and R0 resection rates. We also evaluated technical success, including the endoscopic absence of adenomatous tissue [on inspection with high-definition white light and narrow band imaging (NBI)], average procedure time, amount of water filling the lumen of the large intestine, use of devices such as electrosurgical hemostatic forceps or endoscopic clips, and the rates of adverse events such as bleeding and perforation.

This study was approved by the Institutional Review Board of the Ethics Committee of Chonnam National University Hospital (approval no.: CNUH 2020–333). All patients provided informed consent before the procedure for inclusion in the study. This trial was registered with the International Clinical Trials Registry Platform (No.: KCT0005638, registration date: 2020/11/27).

### 2.2. Procedures and materials

All procedures were performed by an experienced endoscopist (Kim DH) using a high-definition RGB sequential video-endoscopy system (EVIS LUCERA ELITE; Olympus, Tokyo, Japan). Cap-assisted colonoscopy (*CF*-H290I, Olympus) with NBI was performed to confine the margin of the neoplasm under CO_2_ insufflation. Sedative endoscopy was performed in all cases. The patients received an initial intravenous injection of 25 mg pethidine and 0.05 mg/kg midazolam.

Modified UEMR was performed using a water-jet pump (Olympus device), hexagonal snare (Lariat^®^ 00711119, STERIS^tm^, OH, United States), and VAIO 3 (ERBE Co., Ltd., Tubingen, Germany) with a high-frequency generator. The settings of the VAIO 3 were as follows: (1) resection: “precut mode,” Endocut-Q, effect 1, cutting interval 3, cutting duration 3, and power limitation 275 W; (2) vessel ablation using a snare tip or electrosurgical hemostatic forceps (Coagrasper^®^ FD-411UR, Olympus): soft coagulation mode, effect 5.5, and power limit 100 W.

**Figure 1:**
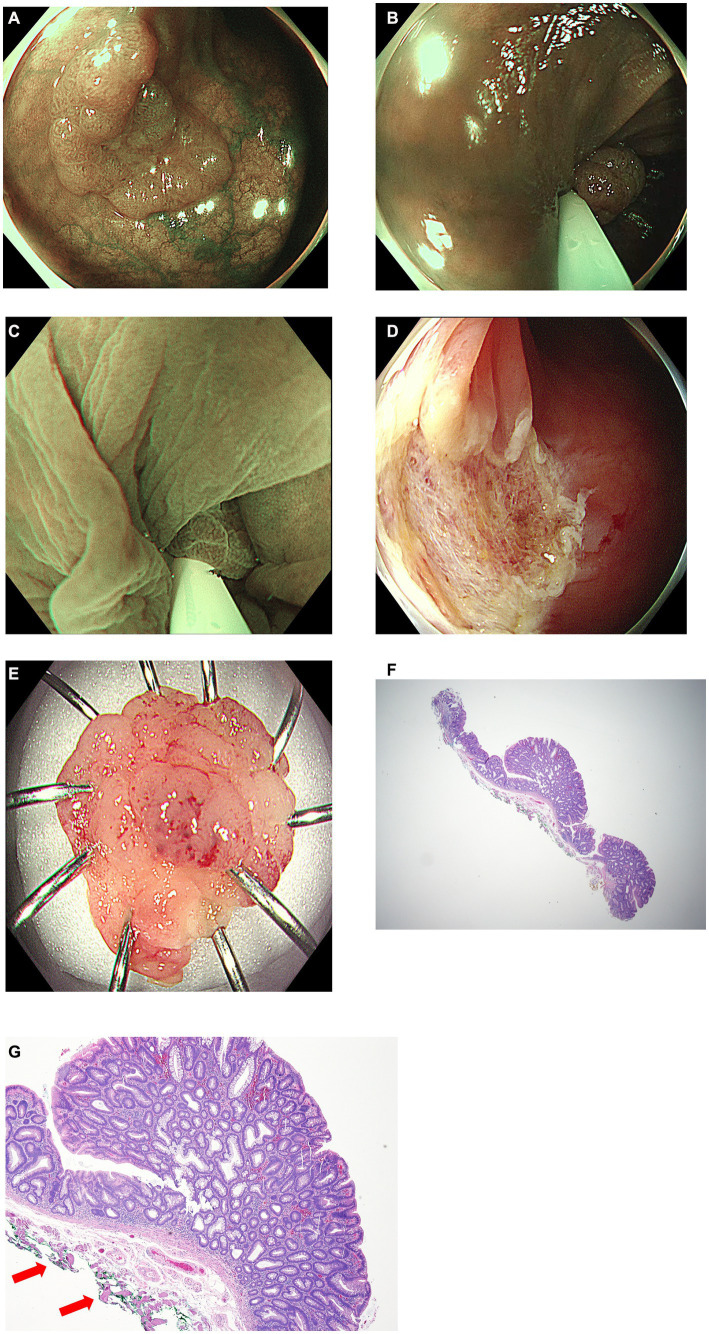
A case of modified underwater endoscopic mucosal resection. **(A)** Endoscopic view of the 16 mm sized sessile colon polyp on transverse colon under narrow-band imaging. **(B)** Snaring the lesion and the surrounding mucosa without injection or water infusion. **(C)** Distilled water was injected using a water-jet pump until the lesion was submerged and excised using electrocautery. **(D)** Endoscopic view of the resected area after endoscopic resection. **(E)** Gross image of the completely removed colon polyp. **(F)** Histology of R0 resection of tubular adenoma with low grade dysplasia using modified underwater endoscopic mucosal resection (×10). **(G)** Red arrows indicate entrapment of muscularis propria in resected specimen (×40).

**Figure 2:**
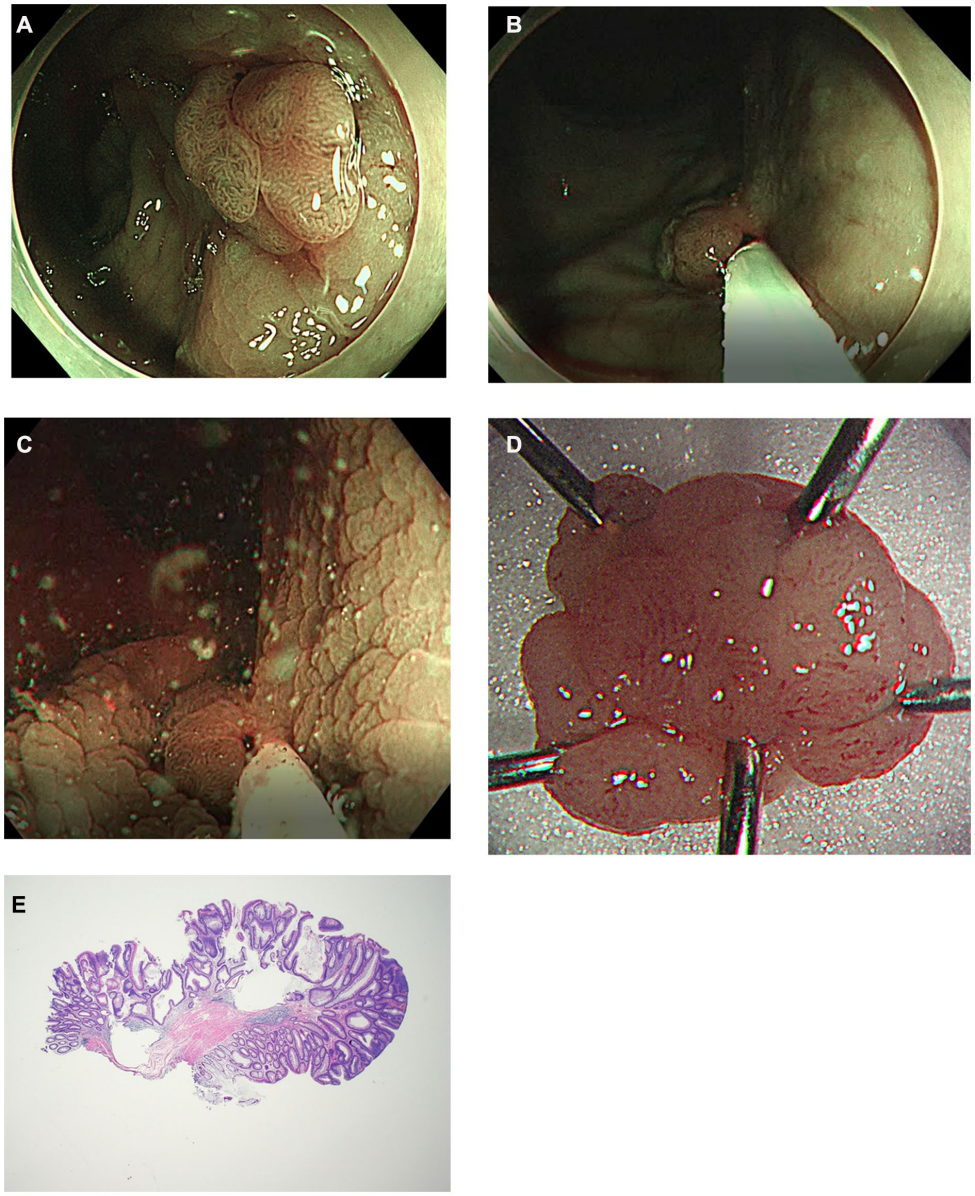
Another case of modified underwater endoscopic mucosal resection. **(A)** Endoscopic view of the 13 mm sized sessile colon polyp on rectum under narrow-band imaging. **(B)** Snaring the lesion and the surrounding mucosa without injection or water infusion. **(C)** Distilled water was injected using a water-jet pump until the lesion was submerged and excised using electrocautery. **(D)** Gross image of the completely removed colon polyp. **(E)** Histology of R0 resection of tubular adenoma with low grade dysplasia using modified underwater endoscopic mucosal resection. No definite evidence of muscularis propria entrapment was noted (×20).

The modified UEMR procedure was carried out as follows: (1) confining the margin of the neoplasm using NBI; (2) snaring the lesion and the surrounding mucosa without injection or water infusion; (3) infusion of distilled water using a water-jet pump until the lesion was submerged; (4) resection of the lesion using electrocautery (precut mode); and (5) vessel ablation was additionally performed using a snare tip or electrosurgical hemostatic forceps (soft-coagulation mode) if immediate bleeding or exposed vessels were observed. The duration of the procedure was measured from the insertion of the snare into the endoscope until the polyps were completely resected and bleeding was controlled (Figures 1, 2 and Supplementary Videos S1, S2).

After resection, the specimens were retrieved and immersed in 10% formalin. Histological diagnosis of the lesion and involvement of the resection margin were evaluated. R0 resection was defined as *en bloc* resection with a histologically confirmed negative resection margin. Non-R0 resection was considered a positive resection margin (R1) or an unclear/indeterminate resection margin (Rx). We also checked the presence or absence of muscle entrapment (muscularis propria) in the resected specimen.

### 2.3. Statistical analyses

Statistical analyses were performed using SPSS version 25.0 (Armonk, NY: IBM Corp.). Continuous data are presented as median (range), while categorical data are presented as absolute and relative frequencies. Continuous variables were analyzed using Student’s *t*-test, and categorical data were examined using Fisher’s exact test or chi-squared test. Statistical significance was set at *p* < 0.05.

## 3. Results

### 3.1. Baseline characteristics

A total of 42 patients with 47 polyps were enrolled in the study. The baseline characteristics of patients and lesions are shown in Table 1. Sex, age, and medication history of the patient were also investigated. Additionally, the location, morphology (15), Japanese NBI Expert Team (JNET) classification (16), and polyp size were evaluated. The median size of the polyp was 12 mm (range, 10–19). Superficial elevated lesions (IIa) (68.1%) were the most common, followed by sessile protruding lesions (Is) (31.9%). The most common JNET classification was 2a (87.2%), followed by 1 (12.8%).

**Table 1:** Baseline characteristics of the study subjects and procedures.

Characteristics	
Patients (n)	42
Treated polyps (n)	47
Sex, Male/Female	30/12
Median Age, years (range)	67 (46–74)
Antithrombotic used, n (%)	1 (2.1)
Antiplatelet, n (%)	4 (8.5)
Anticoagulant, n (%)	0
Hospitalization, n (%)	21 (50.0)
Location, n (%)	
Appendix orifice	2 (4.3)
Cecum	8 (17.0)
Ascending colon	10 (21.3)
Transverse colon	12 (25.5)
Descending colon	6 (12.8)
Sigmoid colon	7 (14.9)
Rectum	2 (4.3)
Morphology, n (%)	
Superficial elevated (IIa)	32 (68.1)
Superficial depressed (IIc)	0
Protruded sessile (Is)	15 (31.9)
Pedunculated (Ip)	0
Median lesion size, mm (range)	12 (10–19)
JNET classification, n (%)	
1	6 (12.8)
2a	41 (87.2)
2b	0
No. of lesions treated per patients, n (%)	
1	39 (92.9)
2	1 (2.4)
3	2 (4.8)
≥4	0

JNET, Japanese NBI expert Team; UEMR, underwater endoscopic mucosal resection.

### 3.2. Procedure-related outcomes

The median duration of the procedure was 71 s (range, 42–607). The median volume of infused fluid was 50 mL (30–130). The rate of R0 resection was 80.9%, and the rate of *en bloc* resection was 97.9%. Technical success was achieved in all cases. The most common histological finding was tubular adenoma (83.0%), followed by SSAP (12.8%). Muscle entrapment was found in 19.1% of the patients. Snare tip ablation and coagrasper ablation were performed in 27.7 and 6.4% of the cases, respectively. Endoscopic clipping was not performed in any patient.

Seven of the polyps (14.9%) were ≥ 15 mm in size, whereas 40 (85.1%) were < 15 mm in size. The median duration of the procedure was 110 s (69–379) and 68 s (42–607) for polyps sized ≥15 mm and < 15 mm (*p* = 0.16), respectively. The median volume of the infused fluid was 70 mL (40–130) and 50 mL (30–120) for polyps sized ≥15 mm and < 15 mm (*p* < 0.01), respectively. R0 resection was observed in 42.9 and 87.5% of patients with polyps sized ≥15 mm and < 15 mm, respectively (*p* < 0.01). *En bloc* resection was observed in 85.7 and 100% of patients with polyps ≥15 mm and < 15 mm in size, respectively (*p* = 0.15). Muscle entrapment was found in 71.4% of patients with polyps sized ≥15 mm and 10% of patients with polyps sized <15 mm (*p* < 0.01). Snare tip ablation was performed in 42.9% of patients with polyps sized >15 mm and 25% with polyps sized <15 mm (*p* = 0.38). Finally, control of bleeding with a coagrasper was performed in 28.6% of patients with polyps sized ≥15 mm and 2.5% with polyps sized <15 mm (*p* = 0.054) (Table 2).

**Table 2:** Procedure-related outcomes.

	Total (*n* = 47)	Polyp size ≥15 mm (*n* = 7)	Polyp size <15 mm (*n* = 40)	*p-*value
R0 resection rate, n (%)	38 (80.9)	3 (42.9)	35 (87.5)	<0.01
R1 resection rate, n (%)	3 (6.4)	2 (28.6)	1 (2.5)	
Rx resection rate, n (%)	6 (12.8)	2 (28.6)	4 (10.0)	
Muscle entrapment, n (%)	9 (19.1)	5 (71.4)	4 (10.0)	<0.01
*En bloc* resection rate, n (%)	46 (97.9)	6 (85.7)	40 (100)	0.15
Piecemeal resection rate, n (%)	1 (2.1)	1 (14.3)	0	
Technical success, n (%)	47 (100)	7 (100)	40 (100)	
Median procedure time, seconds (range)	71 (42–607)	110 (69–379)	68 (42–607)	0.16
Histologic type, n (%)				0.12
Sessile serrated adenoma/polyps	6 (12.8)	2 (28.6)	4 (10.0)	
Tubular adenoma	39 (83.0)	4 (57.1)	35 (87.5)	
Tubulovillous adenoma	2 (4.3)	1 (14.3)	1 (2.5)	
Villous adenoma	0	0	0	
Intramucosal adenocarcinoma	0	0	0	
Submucosal adenocarcinoma	0	0	0	
Fluid infusion (mL), median (range)	50 (30–130)	70 (40–130)	50 (30–120)	<0.01
Snare tip ablation, n (%)	13 (27.7)	3 (42.9)	10 (25.0)	0.38
Coagrasper use, n (%)	3 (6.4)	2 (28.6)	1 (2.5)	0.054
Endoscopic clipping, n (%)	0	0	0	

### 3.3. Adverse events

Of the total subjects, immediate bleeding occurred in 12.8% of patients. Immediate bleeding occurred in 42.9 and 7.5% of patients with polyps sized >15 mm and < 15 mm, respectively (*p* = 0.04). All bleeding events were controlled using a snare tip or a coagrasper. Additionally, no delayed bleeding or perforation occurred, and no other complications were reported (Table 3).

**Table 3:** Adverse events.

Adverse events	Total (*n* = 47)	Polyp size ≥15 mm (*n* = 7)	Polyp size <15 mm (*n* = 40)	*p*-value
Immediate bleeding, n (%)	6 (12.8)	3 (42.9)	3 (7.5)	0.04
Delayed bleeding, n (%)	0	0	0	
Hyponatremia, n (%)	0	0	0	
Perforation, n (%)	0	0	0	

## 4. Discussion

In this study, we removed the intermediated-sized polyp using a polypectomy method that removes the lesion after snaring in air, followed by water infusion, which differs from the previously reported polypectomy method. To the best of our knowledge, this study is the first pilot study to provide evidence for the clinical significance of modified UEMR.

The methods we devised are snaring, water injection, and hot snaring methods, which show the fast endoscopic resection time, which is the advantage of known cold snaring. Additionally, in terms of securing the field of vision, the procedure was successfully performed with minimal impact from bowel preparation. Water infusion was performed after snaring to compensate for damage caused by thermal injury.

Since Binmoeller first devised the UEMR method, it has been successfully used to resect gastrointestinal polyps in various areas (2, 10–12, 17). Conventional UEMR is theoretically based on two main principles (2, 11, 18). First, water immersion decreases the luminal extension force, increases mucosal and submucosal buoyancy, and causes the mucosa, including the lesion, to float upwards into the lumen, while the muscularis propria remains behind the submucosa, which facilitates snaring. Second, underwater resection reduces thermal damage. Submucosal vessels usually remain within the resection wound, as the resection plane is superficial, whereas the submucosal vessels are disrupted in CEMR.

In conventional UEMR, the air is first expelled from the affected area of the lumen, and water is injected until the lumen is completely filled (11). However, it is sometimes challenging to efficiently view the contents underwater, and the addition of a large amount of water carries the inherent risk of electrolyte imbalance (11–13).

The modified UEMR performed in this study had a lesser impact on the floatation of the lesion than the conventional UEMR. This approach decreases the drawbacks associated with conventional UEMR; however, there are concerns regarding the feasibility of grasping a non-floating lesion under water. According to a study by Hirose et al., when a polyp sized 10–14 mm was resected using CSP, an *en bloc* resection rate of 92% and an R0 resection rate of 59.4% were achieved (3). Although there are inherent limitations to a retrospective study, since modified UEMR grasps the lesions in a similar manner as CSP, it is thought that comparable rates of *en bloc* and R0 resection can be achieved. For the resection of large polyps, most studies have reported a technical success rate of approximately 100% using CSP (5, 19, 20). These results suggest that snaring of intermediate-to large-sized polyps may be effective even without water.

According to a recent report on conventional UEMR of intermediated-sized polyps, the rates of *en bloc* resection and R0 resection were 89 and 69%, respectively (2). Additionally, in the CEMR groups, the rates of *en bloc* resection and R0 resection were 50 and 75%, respectively (2). In our study, the rates of *en bloc* resection, R0 resection, and technical success were 97.9, 80.9, and 100%, respectively. The median lesion size (12 mm, range 10–19) was relatively small in our study, which might have resulted in high rates of *en bloc* resection and R0 resection. Considering that the R0 resection rate of lesions larger than 15 mm is 42.9%, which is a large difference from 87.5% observed in lesions smaller than 15 mm in size; it is difficult to determine whether the R0 resection rate is higher in modified UEMR than in conventional UEMR.

In a previous study, the median duration of the UEMR procedure for colon polyps 10–20 mm in size was 165 s (2); however, in our study, the median procedure time was relatively short (71 s). In the same previous study, 200–400 mL of water was infused for most procedures, whereas approximately 50 mL of water was infused for the procedure in our study. This indicates that the modified UEMR method is less burdensome for patients in terms of procedure time and the amount of water added.

Colon endoscopic resection, which includes techniques such as endoscopic mucosal resection (EMR) and endoscopic submucosal dissection (ESD), is a generally safe and effective treatment for the removal of precancerous polyps or early-stage colorectal cancer. However, like any medical procedure, there are associated risks. One potential complication of colon endoscopic resection is perforation, which occurs when a hole is created in the colon wall during the procedure. The perforation rate of colon endoscopic resection can vary based on several factors, including the size and location of the lesion, the technique used, and the experience of the endoscopist. According to a systematic review and meta-analysis of EMR and ESD for colorectal lesions, the incidence of perforation was higher for ESD (4.9%) than for EMR (0.9%) (21). It is important to note that the risk of perforation should be weighed against the potential benefits of colon endoscopic resection in each individual case. Larger and deeper lesions have a higher risk of perforation. The decision to undergo the procedure should be made after careful consideration of the patient’s medical history and the size, location, and characteristics of the lesion to be removed.

In our study, perforation did not occur in any patient. Histological examination revealed muscle capture in 71.4% of lesions larger than 15 mm, whereas muscle capture was observed in only 10% of lesions smaller than 15 mm. Although there were no cases of perforation, it is thought that if modified UEMR is performed on polyps sized ≥15 mm, the muscle capture rate is high, and perforation can be expected if the procedure is repeated for lesions sized ≥15 mm. However, the absence of perforation in all patients indicates that endoscopic energy transfer may be safer than previously believed when performing the procedure without first filling with water (22).

No complications other than immediate bleeding occurred, which was successfully controlled during the procedure. When immediate bleeding or vascular exposure was observed, ablation was mostly performed using a snare tip (soft coagulation mode), which allowed easy control of the bleeding after mucosal resection without the use of additional tools. This led to a decrease in the duration of the procedure. This is also similar to the relatively low bleeding risk of the existing UEMR, suggesting that the modified UEMR also has a low bleeding risk (23). In our study, antithrombotic drugs were used in only one patient (2.1%), and antiplatelet agents were used in four (8.5%). These usage rates are similar to those reported in a previous study on intermediated-sized polyps (CEMR: 4 and 10%, respectively; conventional UEMR 6 and 6%, respectively) (2), and no cases of delayed bleeding occurred. Additional research is needed to conclude whether the procedure can be performed safely in patients using antithrombotics or antiplatelets.

This study was a prospective, observational study conducted at a single center involving a single endoscopist. Owing to the small sample size of this pilot study, it was difficult to confirm the superiority or inferiority of the existing and modified UEMR methods based on these data alone. In addition, since a single endoscopist performed endoscopic resection, the endoscopist’s individual technique of snaring may have an effect on the treatment outcome. As the number of procedures performed increases, perforation that has not occurred until now can be reported. However, through this study, we discovered that UEMR can be performed relatively safely and effectively through the modified method for patients with a poor visual field in an underwater state, which is a disadvantage of UEMR. It was confirmed that snaring without filling the water in air did not increase the risk of perforation or bleeding. Additionally, *en bloc* resection and R0 resection rates also showed good results comparable to UEMR. However, further research is necessary to establish the usefulness of modified UEMR in terms of treatment outcomes and adverse events compared to conventional UEMR or CEMR. A thorough evaluation through a randomized controlled trial is needed in the future.

In conclusion, modified UEMR can be an efficient option in cases where it is challenging to secure a field of vision or it is crucial to prevent electrolyte imbalance, instead of performing a conventional UEMR. Furthermore, careful treatment is required when removing polyps larger than 15 mm.

## Data availability statement

The original contributions presented in the study are included in the article/Supplementary material, further inquiries can be directed to the corresponding authors.

## Ethics statement

The studies involving human participants were reviewed and approved by the Institutional Review Board of the Ethics Committee of Chonnam National University Hospital. The patients/participants provided their written informed consent to participate in this study. Written informed consent was obtained from the individual(s) for the publication of any potentially identifiable images or data included in this article.

## Author contributions

DK, H-SK, and JJ: concept of the study, design, data collection, analysis, interpretation, manuscript drafting and editing, and final approval of the article. S-YP, H-SY, and Y-WJ: data collection, manuscript editing, and review of the manuscript. Y-EJ, D-SM, and S-JK: manuscript review. NK: review of the pathologic result. All authors contributed to the article and approved the submitted version.

## Funding

This study was supported by a grant (BCRI-19024, BCRI-23026, and BCRI-23090) of the Chonnam National University Hospital Biomedical Research Institute.

## Conflict of interest

The authors declare that the research was conducted in the absence of any commercial or financial relationships that could be construed as a potential conflict of interest.

## Publisher’s note

All claims expressed in this article are solely those of the authors and do not necessarily represent those of their affiliated organizations, or those of the publisher, the editors and the reviewers. Any product that may be evaluated in this article, or claim that may be made by its manufacturer, is not guaranteed or endorsed by the publisher.

## Supplementary material

SUPPLEMENTARY VIDEO S1 Video of modified underwater endoscopic resection for a 16 mm sessile colon polyp on the transverse colon.

SUPPLEMENTARY VIDEO S2 Video of modified underwater endoscopic resection for a 13 mm sessile colon polyp on the rectum.
